# Supplementary material for: A phosphorylated transcription factor regulates sterol biosynthesis in Fusarium graminearum
Source: Nat Commun. 2019 Mar 15;10:1228. doi: 10.1038/s41467-019-09145-6 (PMC6420630; doi:10.1038/s41467-019-09145-6)
Supplement: Supplementary file 1 — Supplementary Information [file 41467_2019_9145_MOESM1_ESM.pdf]

A phosphorylated transcription factor regulates sterol biosynthesis in *Fusarium graminearum*

Zunyong Liu et al.

Supplementary Information

Supplementary information

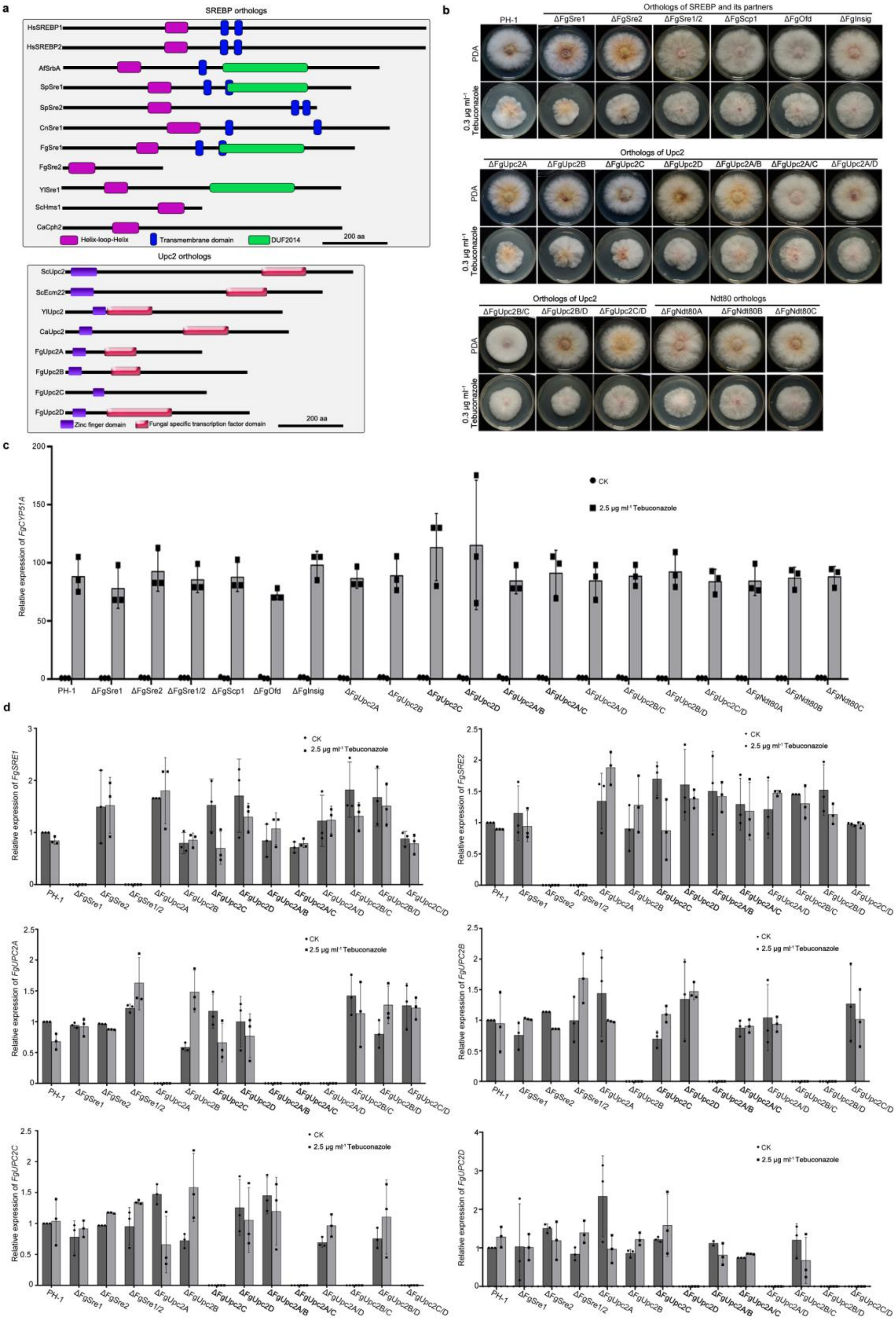

Supplementary Figure 1 | The orthologs of SREBP and Upc2 in *F.*

***graminearum*are not involved in regulating sterol biosynthesis. (a)** Protein domain structures of SREBP and Upc2 orthologs from different eukaryotes were analyzed with the SMART protein database (http://smart.embl-heidelberg.de) and the NBCI protein database (https://blast.ncbi.nlm.nih.gov/Blast.cgi). SREBP orthologs contain HsSREBP-1 and -2 from *H.sapiens*, AfSrbA from *A. fumigatus*, SpSre-1 and -2 from *S. pombe*, CnSre1 from *C. neoformans*, FgSre-1 and -2 from *F. graminearum*, YlSre1 from *Y. lipolytica*, ScHms1 from *S. cerevisiae*, and CaCph2 from *C. albicans*. Upc2 orthologs include ScUpc2 and ScEcm22 from *S. cerevisiae*, YlUpc2 from *Y. lipolytica*, CaUpc2 from *C. albicans* and FgUpc2-A, -B, -C and -D from *F. graminearum*. **(b)** The *F. graminearum* mutants of SREBP and Upc2 orthologs, SREBP's partners and three Ndt80 paralogs did not show increased sensitivity to azole compounds. A 5-mm mycelial plug of each strain was inoculated on PDA alone or supplemented with 0.3 μg ml^{-1} tebuconazole, and then incubated at 25°C for 3 days. **(c)** SREBP and Upc2 orthologs, SREBP's partners and three Ndt80 paralogsdid not regulate the transcription of *FgCYP51A* under tebuconazole treatment. Each strain was cultured in YEPD for 36 hours, and then treated with 2.5 μg ml^{-1} tebuconazole for 6 hours. The expression level of *FgCYP51A* in PH-1 without treatment was referred to 1 and the *FgACTIN* gene was used as the internal control for normalization. **(d)** The transcription of each SREBP or Upc2 ortholog was not changed by tebuconazole treatment or deletion of another SREBP or Upc2 ortholog. Each strain was cultured in YEPD for 36 hours, and then treated with 2.5 μg ml^{-1} tebuconazole for 6 hours. The expression level of each genein PH-1 without treatment was referred to 1 and the

FgACTIN gene was used as the internal control for normalization.

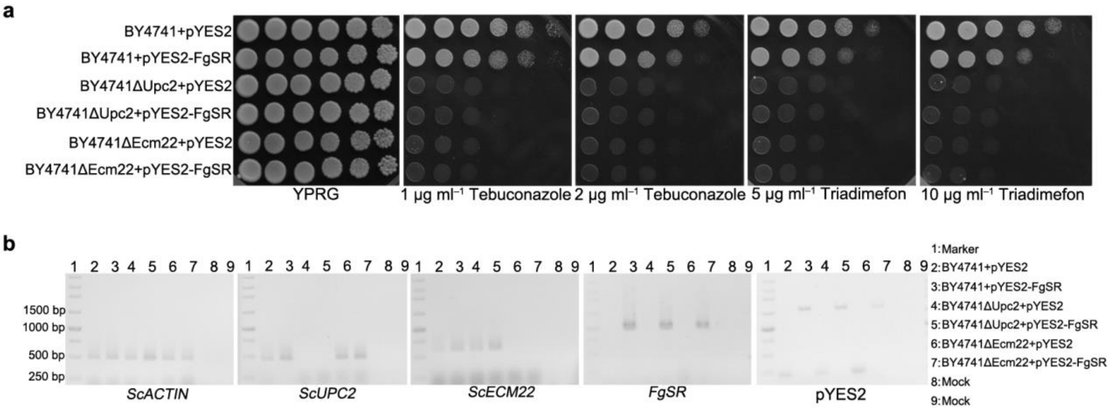

Supplementary Figure 2 | FgSR is unable to complement sensitivity to azole compounds in the yeast Upc2or Ecm22 mutant. (a) Cells of the transformants containing pYES2 or pYES2-FgSR were spotted onto YPRG supplemented with tebuconazole at 1, or 2 μg ml^{-1}, or triadimefon at 5 or 10 μg ml^{-1}. In addition, the wild-type strain BY4741 transformed withempty pYES2 was used as a control. **(b)** PCR verification for each transformants. The insertion of *FgSR* or pYES2 in each transformant was amplified with primer pairs shown in Table S1. Yeast Upc2 or Ecm22 mutants were confirmed by amplifying the *ScACTIN*, *ScUPC2* and *ScECM22* genes using primer pairs shown in Table S1. The gene detected or vector pYES2 is indicated at the bottom of each panel.

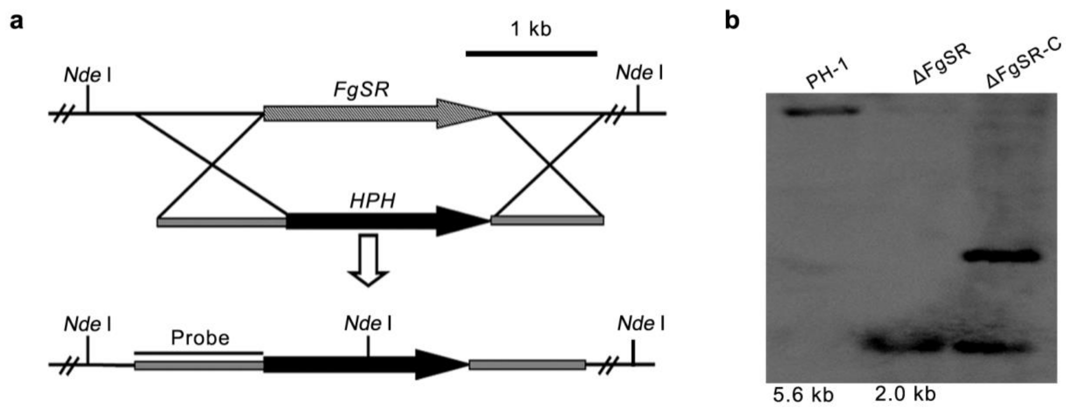

Supplementary Figure 3 | Construction and identification of FgSR gene deletion- and complemented mutants. (a) Gene replacement strategy for the FgSR deletion mutant. The hygromycin resistance cassette (HPH) is denoted by the large black arrow. **(b)** Southern blot hybridization analysis of the wild-type strain PH-1, FgSR deletion mutant ΔFgSR and the complemented transformant ΔFgSR-C using a 959-bp FgSR fragment as a probe.

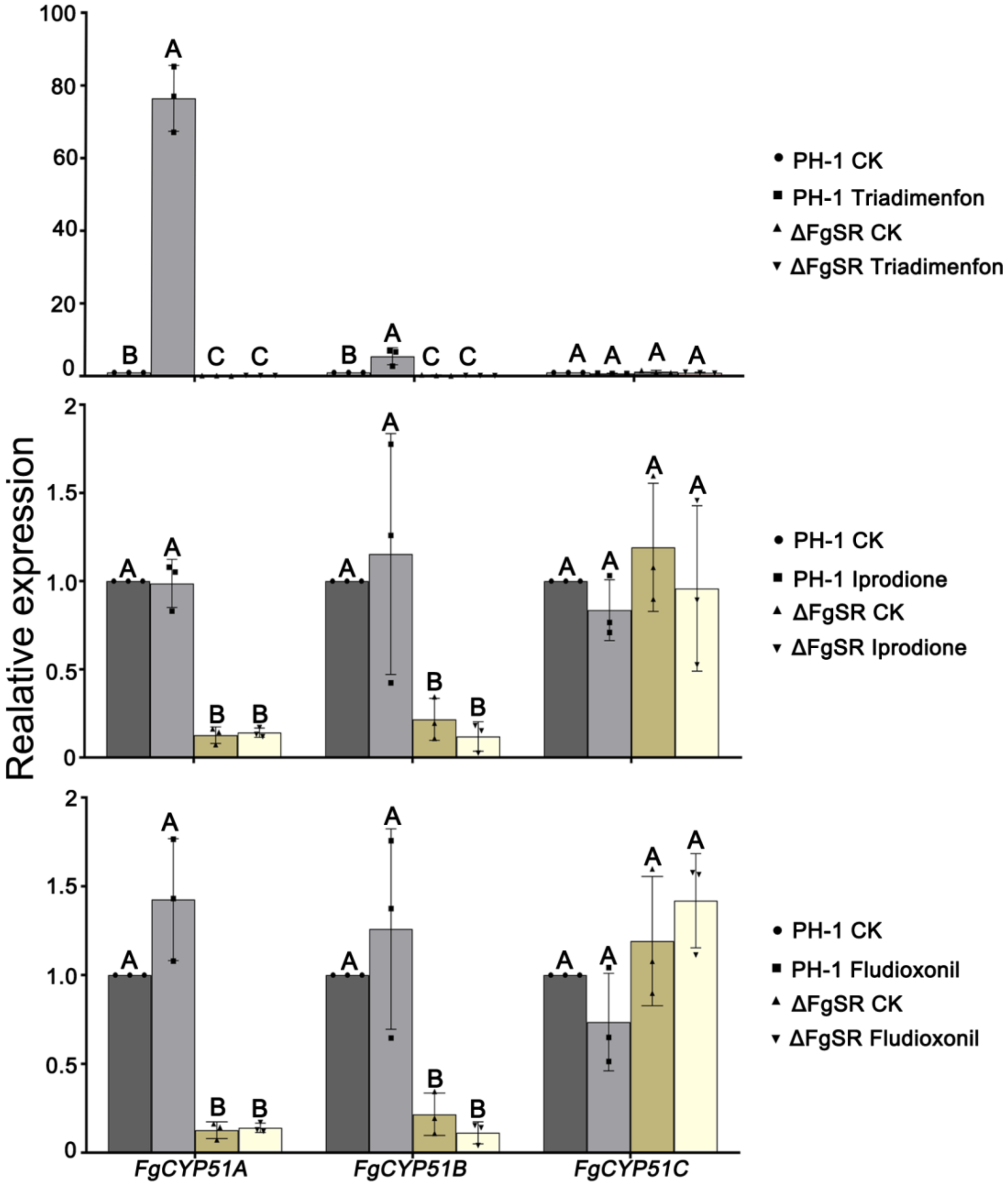

Supplementary Figure 4 | The expression of *FgCYP51A* and *FgCYP51B* is induced by treatment with tridiamefon but not iprodione and fuldioxonil. Each strain was cultured in YEPD for 36 hours, then treated with 10 μg ml^{-1} tridiamenfon (top), 25 μg ml^{-1} iprodione (middle) or 2 μg ml^{-1} fludioxonil (bottom) for 6 hours. The expression level of each *FgCYP51* in PH-1 without treatment was set to 1 (indicated by red line) and the *FgACTIN* gene was determined as the internal control for normalization.Data presented are the mean ± s.d. (n = 3). Bars followed by the

same letter are not significantly different according to a LSD test at $P = 0.01$.

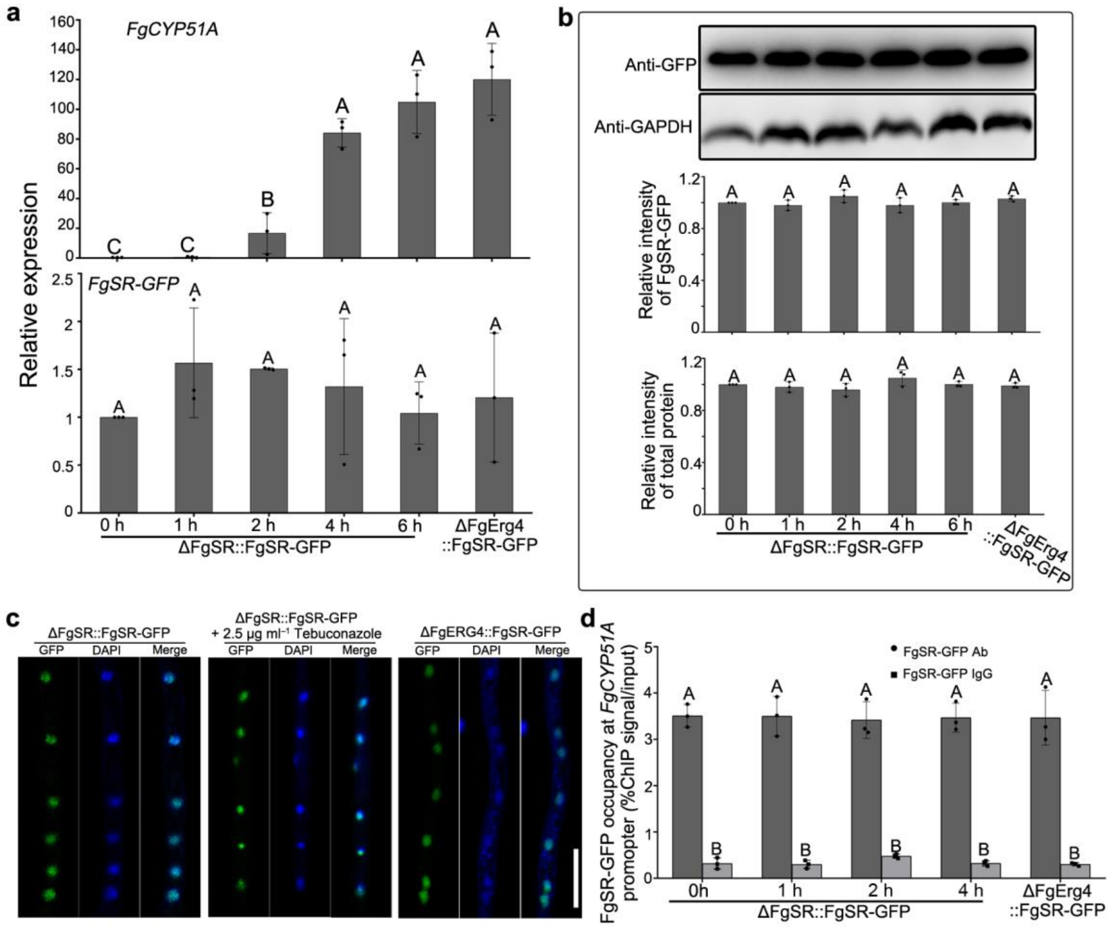

Supplementary Figure 5 | The transcription, protein accumulation, subcellular localization, and enrichment at the *FgCYP51A* promoter of FgSR were not altered under ergosterol-depleted conditions. (a) Comparisons of the transcript levels of *FgCYP51A* (top panel) and *FgSR-GFP* (lower panel) among various treatments. The strain ΔFgSR::FgSR-GFP cultured in YEPD for 36 h and then treated with 2.5 μg ml^{-1} tebuconazole for 0, 1, 2, 4 and 6 hours. ΔFgErg4 was used to mimic the ergosterol-absent conditions. The expression level of each gene in PH-1 without treatment was set to the value 1. **(b)** The protein content of FgSR-GFP was not changed by tebuconazole treatment. The western blot bands were detected with the monoclonal anti-GFP antibody and the monoclonal anti-GAPDH antibody as a reference. The intensities of the western blot bands were quantified with the program

IMAGE QUANT TL. The intensities of the FgSR-GFP and GAPDH without treatment were set to the value 1. **(c)** FgSR-GFP was constitutively present in the nucleus of ΔFgSR::FgSR-GFP before (left panel) or after tebuconazole treatment (middle panel). FgSR-GFP was also localized inthe nucleus of the ΔFgErg4:: FgSR-GFP mutant (right panel). Bar = 10 μm. **(d)** The enrichment of FgSR-GFP at the promoter of *FgCYP51A* in ΔFgSR::FgSR-GFP after treatment with tebuconazole for 0 to 4 hour, and in ΔFgErg4:: FgSR-GFP. ChIP- and input-DNA samples were quantified by PCR using the primer pair A2 indicated in Fig. 2b. Data presented are the mean ± s.d. (n = 3). Bars followed by the same letter are not significantly different according to a LSD test at P = 0.01.

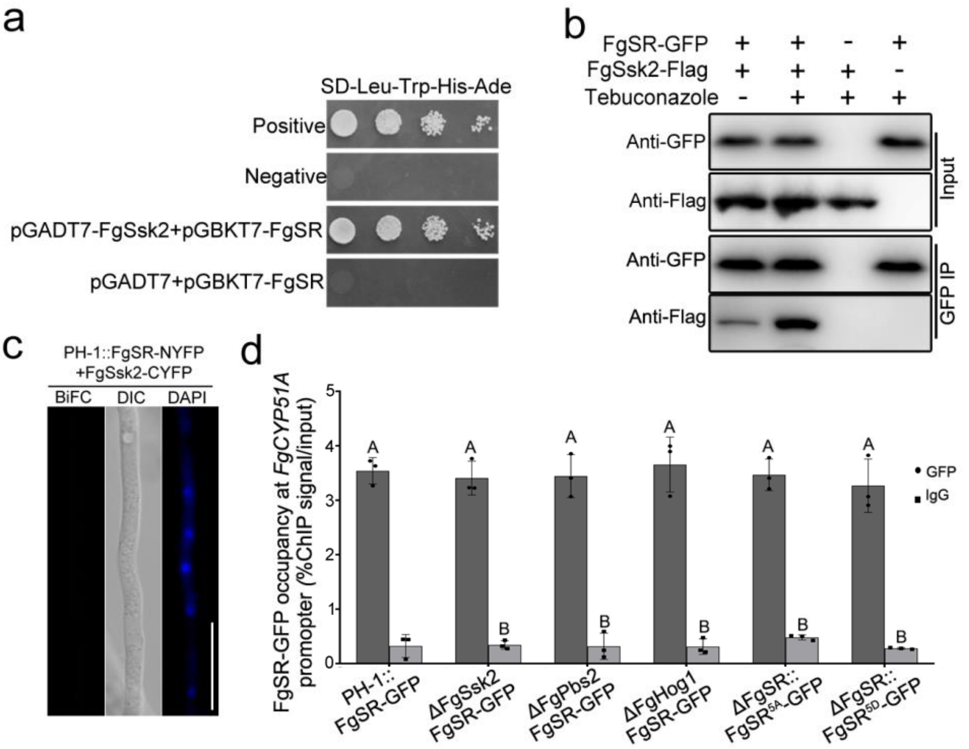

Supplementary Figure 6 | FgSsk2 interacts with FgSR, but does not change the enrichment of FgSR at the *FgCYP51A* promoter. (a) FgSsk2 interacts with FgSR in the yeast two-hybrid (Y2H) assay. Serial dilutions of yeast cells (cells ml^{-1}) transferred with the bait and prey constructs indicated in the figure were assayed for growth on SD-Leu-Trp-His-Ade plates. **(b)** FgSsk2 interacts with FgSR in the co-immunoprecipitation (Co-IP) assay and tebuconazole treatment enhanced the interaction. The strains were treated with or without tebuconazole for 2 hours after incubated in YEPD for 36 hours. Total proteins (input) extracted from the strain containing a pair of constructs or a single construct were subjected to SDS-PAGE and immunoblots were incubated with the monoclonal anti-Flag and monoclonal anti-GFP antibodies as indicated. Then, each protein sample was pulled down using anti-GFP agarose and further detected with the monoclonal anti-GFP and anti-Flag antibodies. **(c)** FgSsk2 does not interact with FgSR under the treatment with 2.5 μg ml^{-1}

tebuconazole by the bimolecular fluorescence complementation (BiFC) assay. YFP signals were observed using confocal microscopy. Bar = 10 μm. **(d)** The enrichment of FgSR-GFP at the *FgCYP51A* promoter of each strain. ChIP- and input-DNA samples were quantified by quantitative PCR assays with the primer pair A2 indicated in Fig. 2b, and rabbit IgG was used as a control. Data presented are the mean ± s.d. (n = 3). Bars followed by the same letter are not significantly different according to a LSD test at $P = 0.01$.

a

Amino site	Context	Score	Kinase	Answer
82 T	SAPRTPLTR	0.608	p38MAPK	YES
92 S	RAPSSPARA	0.577	p38MAPK	YES
102 S	RPNPSPPTS	0.535	p38MAPK	YES
217 T	GAGITPENA	0.506	p38MAPK	YES
305 S	LNPRSPDSF	0.514	p38MAPK	YES

b

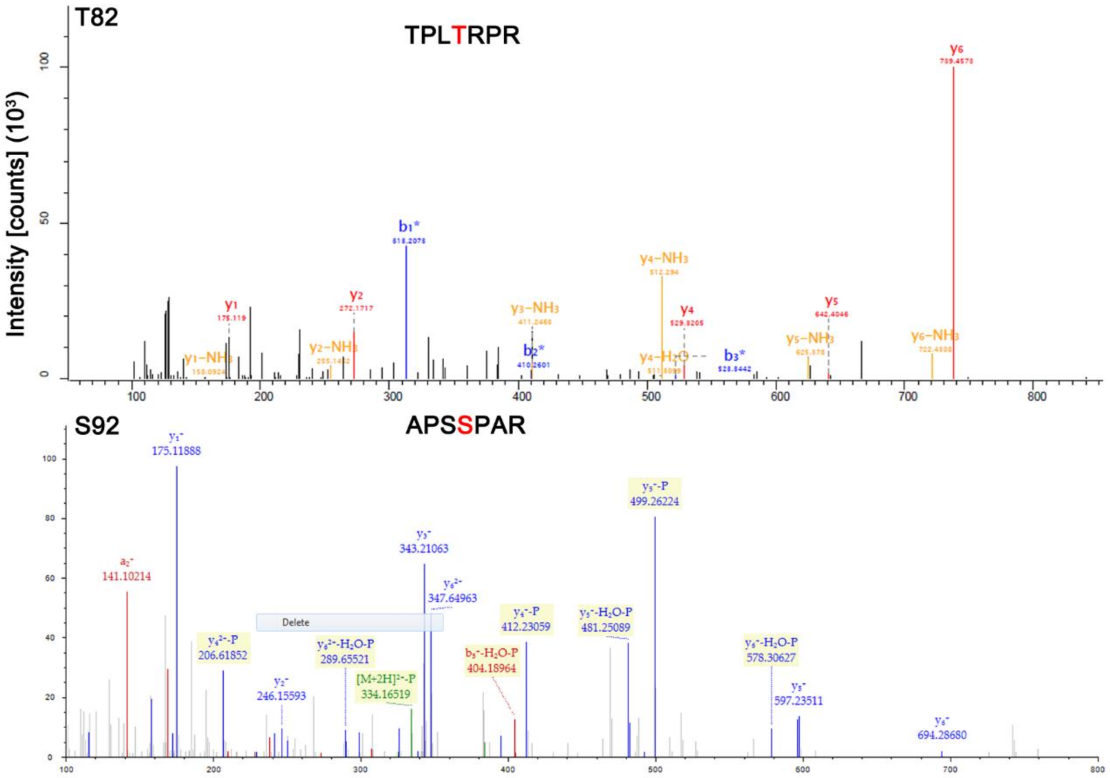

c

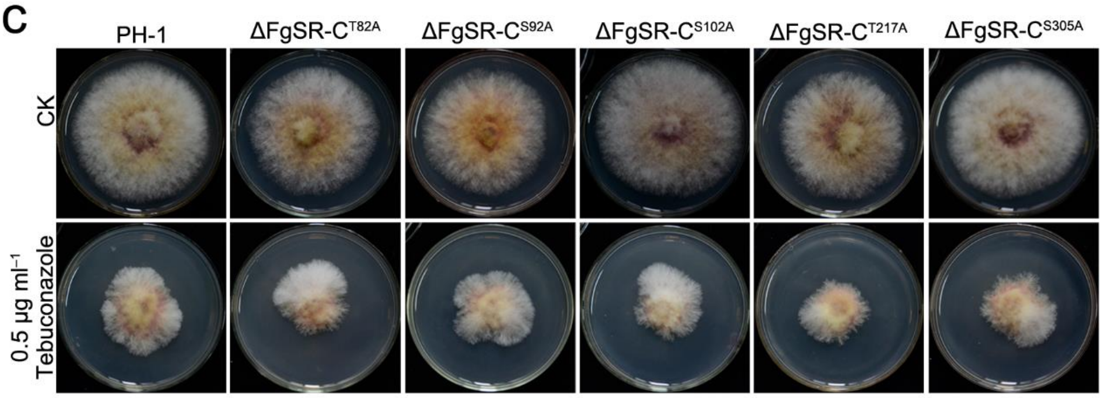

Supplementary Figure 7 | Identification and functional analysis of putative phosphorylation sites of FgSR. (a) The five phosphorylated residues Thr82, Ser92,

Ser102, Thr217 and Ser305 in FgSR predicted using NetPhos 3.1 Server (http://www.cbs.dtu.dk/services/NetPhos/). **(b)** The two phosphorylated residues Thr82 and Ser92 in FgSR identified by mass spectrometry assay. **(c)** The mutants carrying individual phosphorylation site mutated from S/T to Adid not exhibit elevated sensitivity to tebuconazole. A 5-mm mycelial plug of each strain was inoculated on PDA alone or supplemented with 0.5 μg ml^{-1} tebuconazole, and then incubated at 25°C.

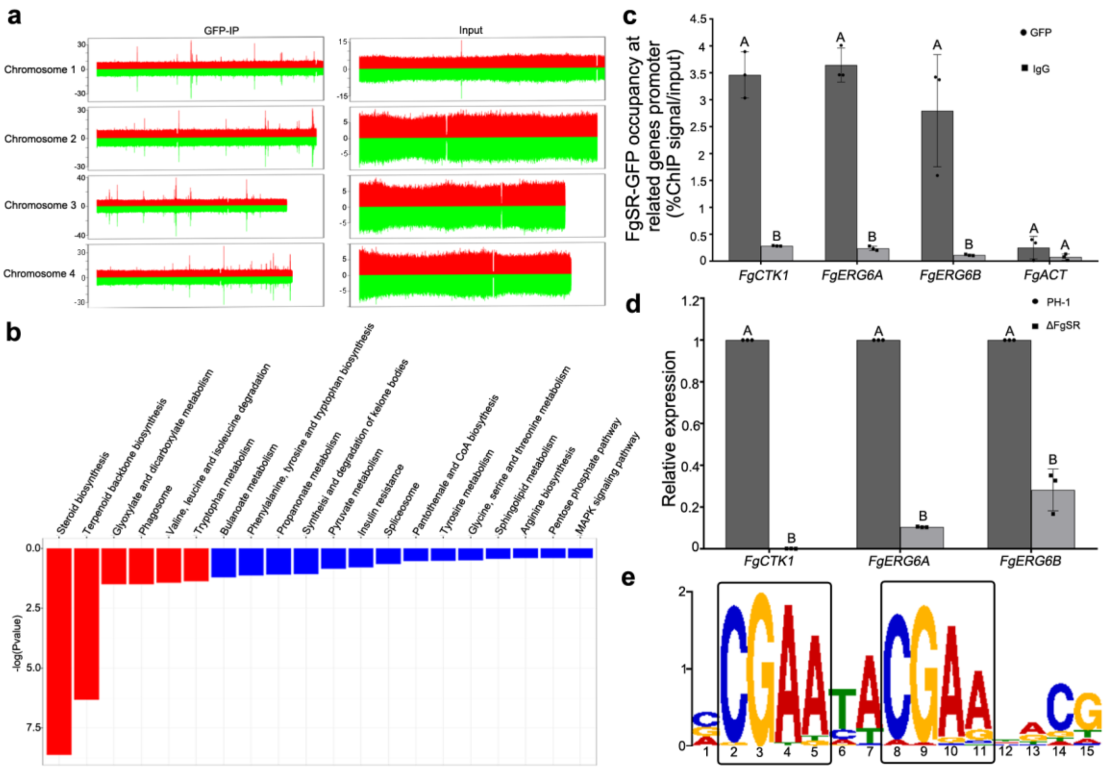

Supplementary Figure 8 | Genome-wide ChIP-Seq analysis for identification of target genes of FgSR. **(a)** Browser shot of FgSR binding sites on all four chromosomes of *F. graminearum*. **(b)** Enrichment of FgSR-binding genes on the Kyoto Encyclopedia of Genes and Genomes (KEGG) pathways. **(c)** ChIP-qPCR verified the enrichment of FgSR at the promoters of *FgCTK1*, *FgERG6A* and *FgERG6B* that are randomly selected according to the ChIP-Seq data. Rabbit IgG was used as a control and *FgACTIN* was detected as a negative control. **(d)** Comparisons of expression of each gene in the wild type and the ΔFgSR mutant after growth in PDB for 1 day. The expression level of each gene in the wild type was set to 1. Data presented are the mean ± s.d. ($n = 3$). Bars followed by the same letter are not significantly different according to a LSD test at $P = 0.01$. **(e)** The putative motif was identified from the ChIP-Seq data sets.

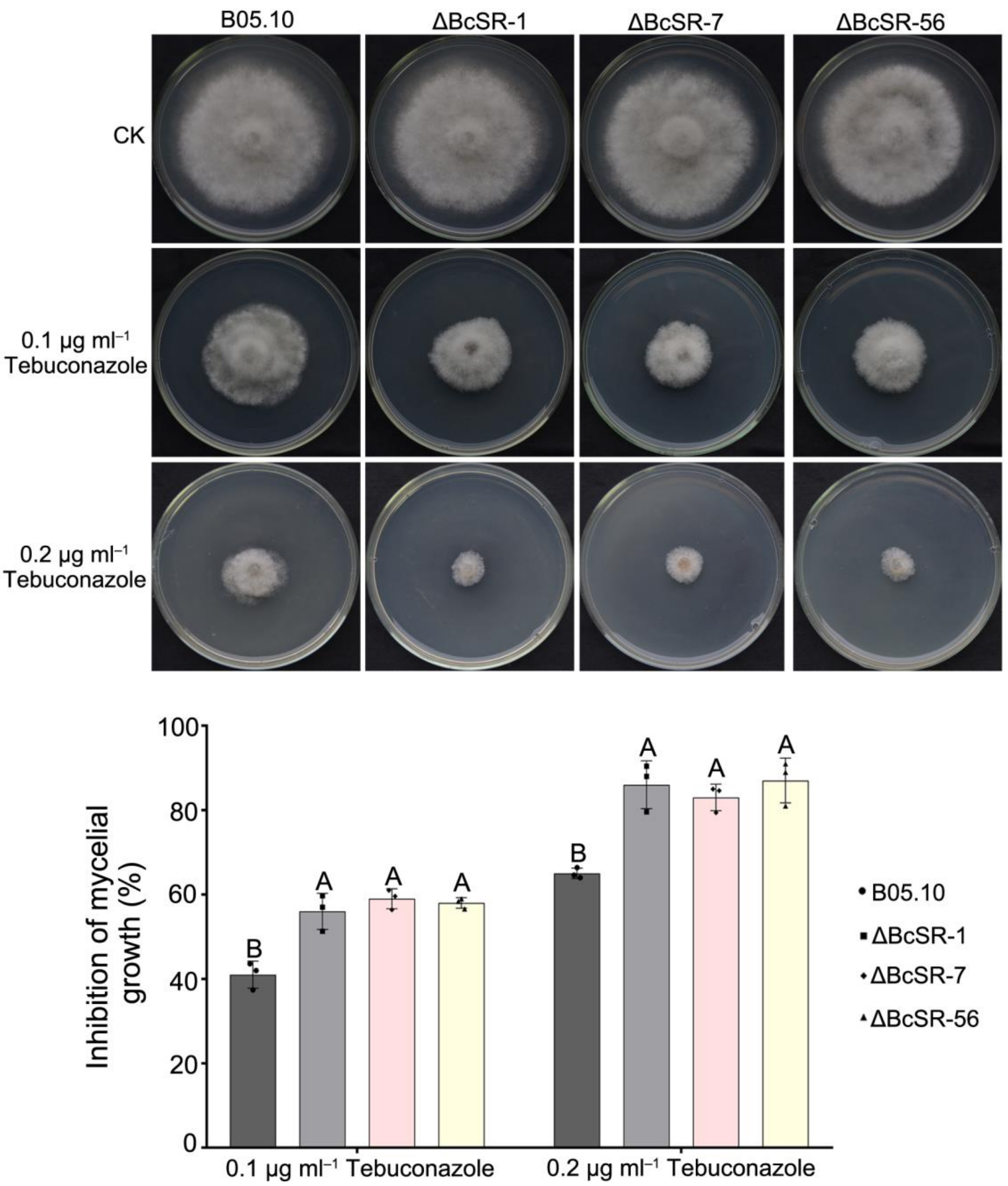

Supplementary Figure 9 | Deletion of the FgSR homolog in the *Leotiomycetes* fungus *Botrytis cinerea* leads to increased sensitivity to tebuconazole. A 5-mm mycelial plug of each strain was inoculated on PDA alone or supplemented with tebuconazole at 0.1 or 0.2 μg ml^{-1}, and then incubated at 25°C for 3 days. Mycelial growth inhibition of each strain under each treatment.Data presented are the mean ± s.d. (n = 3). Bars followed by the same letter are not significantly different according to a LSD test at P = 0.01.

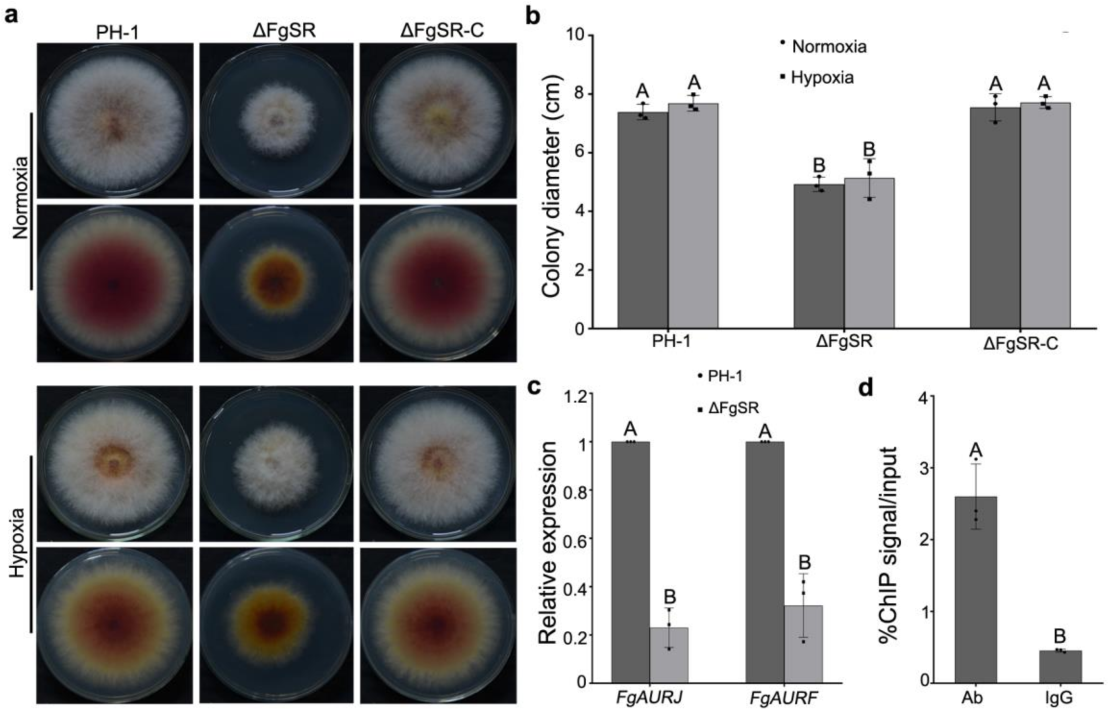

Supplementary Figure 10 | FgSR regulates the biosynthesis of aurofusarin. (a) Mycelial growth of the wild type, ΔFgSR, and ΔFgSR-C incubated on PDA at 25°C for 3 days in normoxia or hypoxia (1% O_2 and 5% CO_2). **(b)** Colony diameter of each strain cultured on PDA in normoxia and hypoxia. **(c)** Relative expression levels of aurofusarin biosynthesis genes *FgAURJ* and *FgAURF* in the wild type and ΔFgSR. The expression level of each gene in the wild type was referred to 1. **(d)** The enrichment of FgSR-GFP at the common promoter of *FgAURJ* and *FgAURF*. Rabbit IgG was used as a control. Data presented are the mean ± s.d. (n = 3). Bars followed by the same letter are not significantly different according to a LSD test at P = 0.01.

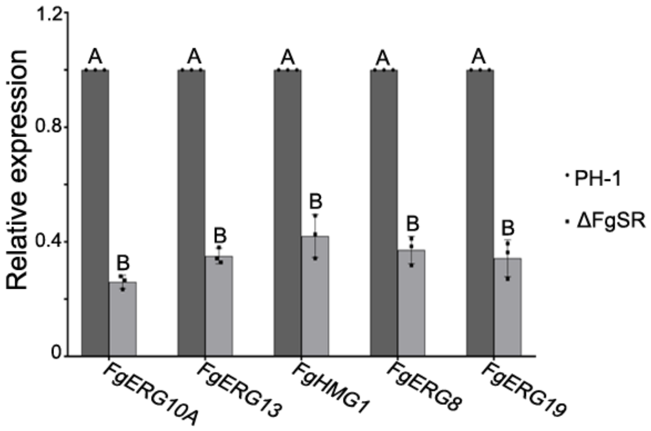

Supplementary Figure 11 | The expression of five isoprenoid biosynthesis genes was down-regulated in the ΔFgSR mutant under the trichothecene biosynthesis inducing conditions. The wild type and the ΔFgSR mutant were cultured in liquid trichothecene biosynthesis inducing (TBI) medium in the dark at 28°C for two days on a shaker (150 rpm). The expression level of each gene in the wild type was set to 1. Data presented are the mean ± s.d. (n = 3). Bars followed by the same letter are not significantly different according to a LSD test at P = 0.01.

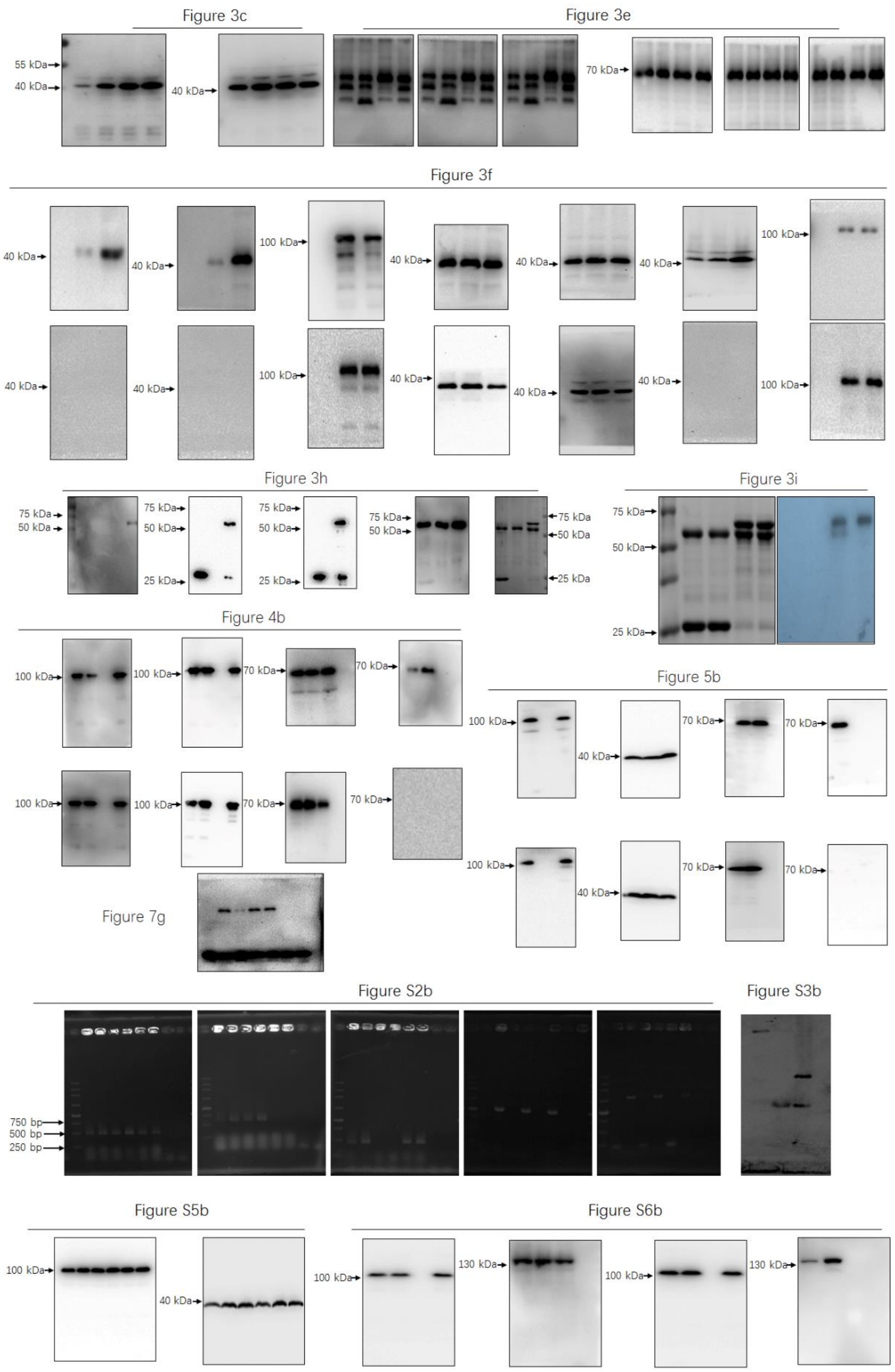

Supplementary Figure 12 | Original full length images for immunoblots and gels. The figures associated with each immunoblot or gel image was indicated above the images, molecular weight markers are indicated in the image.

Supplementary Table 1 | The putative *cis*-elements in promoters of *ERG11* orthologs from 27 organisms.

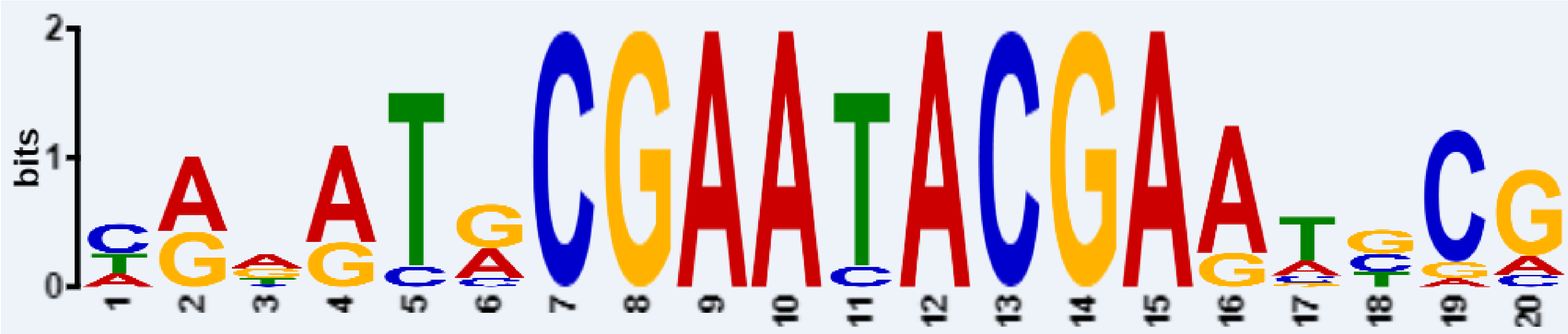

Species	*ERG11* homologues	If containing *cis-* element	p-value	The putative sterol regulatory bond by FgSR in *ERG11* promoters
Saccharomyces cerevisiae	*ERG11*	N		
Candida glabrata	*CAGL0E04334g*	N		
Candida albicans	*ERG11*	N		
Candida parapsilosis	*CPAR2_303740*	N		
Yarrowia lopolytica	*YALI0_B05126g*	N		
Fusarium verticillioides	*FVEG_01123*	Y	5.07e-9	TATTGGCAAG CAGATAAGAATGCGAAAAAG GAGGGATGGA
	FVEG_12077	Y	4.60e-11	TTCAGAAGGT CAAGTACGAATACGAATGCG ACAACCATCC
	FVEG_12391	N		
Fusarium_fujikuroi	*FFUJ_09534*	Y	4.60e-11	CTTCAGATGC CAAGTACGAATACGAATGCG AACAACCCTC
	FFUJ_01179	Y	5.07e-9	TATTGGCGGG CAGATAAGAATGCGAAAAAG GAGGGATGGA
Fusarium oxysporum f.	*FOXG_00394*	Y	5.07e-9	TATTGGCGGG CAGATAAGAATGCGAAAAAG GAGGGATGGA

sp. lycopersici	*FOXG_11545*	Y	4.60e-11	TTCACAAGGC	CAAGTACGAATACGAATGCG	AACAACCTTC
	FOXG_13138	N				
Fusarium graminearum	*FGSG_04092*	Y	1.02e-10	CCTGTCAAGA	CACGTGCGAATACGAATGCG	AAAACTTCAA
	FGSG_01000	Y	3.76e-10	GTTCTAGAAG	TAGATGCGAATACGAATTCG	GTGCCTCACA
	FGSG_11024	Y	6.77e-8	TCGATTAAAT	CGGATGCGAATACGAGATGA	GTCAGCCCTA
Trichoderma reesei	*TRIREDRAFT_75468*	Y	5.44e-8	GCGGAGAATT	TGCGGGCGAATGCGAATACG	TCCCTCTCTC
Colletotrichum graminicola	*GLRG_01612*	Y	4.07e-8	ATGAGGGTCC	CGGATACGAATACGAACGGC	CCGCCATTGG
Neurospora crassa	*NCU02624*	Y	3.26e-9	CGTCCAACCA	AAAATCCGAATACGAATGCG	TTGCCCCTGC
Podospora anserine	*PODANS_1_4470*	Y	1.25e-9	AGGTACCGTG	CGAATGCGAATACGAAACCG	CTCTTGGGGA
Chaetomium globosum	*CHGG_01652*	Y	3.76e-10	GTTCTAGAAG	TAGATGCGAATACGAATTCG	GTGCCTCACA
Magnaporthe grisea	*MGG04628*	Y	3.83e-8	GGCCCCAAAC	AGAACACGAATACGAGTGCG	AGGCTCGGAT
	MGG04432	Y	3.91e-9	GGCGTTGAAC	TGAATGCGAATACGAATTAG	AGAAACCGAA
Botrytis cinerea	*Bccyp51*	Y	1.25e-9	AAATTTACCT	CAGGTACGAACACGAAATCG	GTGTGGCCAA
Sclerotina sclerotiorum	*SS1G_04805*	Y	7.71e-9	GCGAGAATTT	AAGGTACGAACACGAAATCG	CGGTGACCAA
Aspergillus fumigates	*AFUA_4G06890*	N				
	AFUA_7G03740	N				
Penicillium digitatum	*PDIG_70830*	N				
	PDIG_14340	N				
Histoplasma capsulatum	*HCBG_09081*	N				
	HCBG_00405	N				
Paracoccidioides brasiliensis	*ACO22_03253*	N				
Schizosaccharomyces	*ERG11*	N				

prombe				
Cryptococcus neoformans	*CNAG_00040*	N		
Homo sapiens	*CYP51A1P1*	N		
	CYP51A1P2	N		
Caenorhabditis elegans	cyp_25A1	N		
	cyp_25A2	N		
	cyp_25A3	N		
Drosophila melanogaster	Cyp6d4	N		
	Cyp6a13	N		
