# Supplementary material for: A phosphorylated transcription factor regulates sterol biosynthesis in Fusarium graminearum
Source: Nat Commun. 2019 Mar 15;10:1228. doi: 10.1038/s41467-019-09145-6 (PMC6420630; doi:10.1038/s41467-019-09145-6)
Supplement: Supplementary file 3 — Description of Additional Supplementary Files [file 41467_2019_9145_MOESM3_ESM.pdf]

**Description of Additional Supplementary Files**

File Name: Supplementary Data 1
Description: A list of putative FgSR-interacting proteins identified by yeast two-hybrid screens.

File Name: Supplementary Data 2
Description: Putative target genes of FgSR identified by ChIP-Seq.

File Name: Supplementary Data 3
Description: The differentially expressed genes in the FgSR deletion mutant with RNA-seq analysis.

File Name: Supplementary Data 4
Description: A list of PCR primers used in this study and their relevant characteristics.
