# Supplementary material for: A phosphorylated transcription factor regulates sterol biosynthesis in Fusarium graminearum
Source: Nat Commun. 2019 Mar 15;10:1228. doi: 10.1038/s41467-019-09145-6 (PMC6420630; doi:10.1038/s41467-019-09145-6)
Supplement: Supplementary file 8 — Reporting Summary [file 41467_2019_9145_MOESM8_ESM.pdf]

nature research

Corresponding author(s): Zhonghua Ma
Yanni Yin

Last updated by author(s): Feb 4, 2019

Reporting Summary

Nature Research wishes to improve the reproducibility of the work that we publish. This form provides structure for consistency and transparency in reporting. For further information on Nature Research policies, see Authors & Referees and the Editorial Policy Checklist.

Statistics

For all statistical analyses, confirm that the following items are present in the figure legend, table legend, main text, or Methods section.

n/a	Confirmed	
☐	☒	The exact sample size (*n*) for each experimental group/condition, given as a discrete number and unit of measurement
☐	☒	A statement on whether measurements were taken from distinct samples or whether the same sample was measured repeatedly
☐	☒	The statistical test(s) used AND whether they are one- or two-sided<br>*Only common tests should be described solely by name; describe more complex techniques in the Methods section.*
☐	☒	A description of all covariates tested
☐	☒	A description of any assumptions or corrections, such as tests of normality and adjustment for multiple comparisons
☐	☒	A full description of the statistical parameters including central tendency (e.g. means) or other basic estimates (e.g. regression coefficient) AND variation (e.g. standard deviation) or associated estimates of uncertainty (e.g. confidence intervals)
☐	☒	For null hypothesis testing, the test statistic (e.g. *F*, *t*, *r*) with confidence intervals, effect sizes, degrees of freedom and *P* value noted<br>*Give P values as exact values whenever suitable.*
☐	☒	For Bayesian analysis, information on the choice of priors and Markov chain Monte Carlo settings
☐	☒	For hierarchical and complex designs, identification of the appropriate level for tests and full reporting of outcomes
☐	☒	Estimates of effect sizes (e.g. Cohen's *d*, Pearson's *r*), indicating how they were calculated

Our web collection on statistics for biologists contains articles on many of the points above.

Software and code

Policy information about availability of computer code

Data collection	Not Applicable.
Data analysis	Not Applicable.

For manuscripts utilizing custom algorithms or software that are central to the research but not yet described in published literature, software must be made available to editors/reviewers. We strongly encourage code deposition in a community repository (e.g. GitHub). See the Nature Research guidelines for submitting code & software for further information.

Data

Policy information about availability of data

All manuscripts must include a data availability statement. This statement should provide the following information, where applicable:

- Accession codes, unique identifiers, or web links for publicly available datasets
- A list of figures that have associated raw data
- A description of any restrictions on data availability

Data availability. The ChIP-Seq data has been deposited in the NCBI BioProject database with accession code PRJNA520738. Other relevant data supporting the findings of the study are available in this article and its Supplementary Information files.

Field-specific reporting

Please select the one below that is the best fit for your research. If you are not sure, read the appropriate sections before making your selection.

☒ Life sciences ☐ Behavioural & social sciences ☐ Ecological, evolutionary & environmental sciences

Life sciences study design

All studies must disclose on these points even when the disclosure is negative.

Sample size	Not applicable
Data exclusions	Not applicable
Replication	Not applicable
Randomization	Not applicable
Blinding	Not applicable

Reporting for specific materials, systems and methods

We require information from authors about some types of materials, experimental systems and methods used in many studies. Here, indicate whether each material, system or method listed is relevant to your study. If you are not sure if a list item applies to your research, read the appropriate section before selecting a response.

Materials & experimental systems

n/a	Involved in the study	
☐	☒	Antibodies
☒	☐	Eukaryotic cell lines
☒	☐	Palaeontology
☒	☐	Animals and other organisms
☒	☐	Human research participants
☒	☐	Clinical data

Methods

n/a	Involved in the study	
☐	☒	ChIP-seq
☒	☐	Flow cytometry
☒	☐	MRI-based neuroimaging

Antibodies

Antibodies used	anti-Flag antibody (A9044, Sigma, St. Louis, MO); anti-GFP (ab32146, Abcam, Cambridge, UK); anti-P-p-38 (#9211, Cell Signaling Technology Inc., Boston, MA, USA) andibody; anti-Hog (sc-165978, Santa Cruz, CA, USA) antibody; anti-GAPDH (EM1101, Hangzhou HuaAn Biotechnology co., Ltd.) antiboy; anti-GFP ab290 (Abcam, Cambridge, UK) antibody; anti-H3 (ab1791, Abcam, Cambridge, UKn) antibody.
Validation	anti-Flag antibody (A9044, Sigma, St. Louis, MO); anti-GFP (ab32146, Abcam, Cambridge, UK); anti-P-p-38 (#9211, Cell Signaling Technology Inc., Boston, MA, USA) andibody; anti-Hog (sc-165978, Santa Cruz, CA, USA) antibody; anti-GAPDH (EM1101, Hangzhou HuaAn Biotechnology co., Ltd.) antiboy; anti-GFP ab290 (Abcam, Cambridge, UK) antibody; anti-H3 (ab1791, Abcam, Cambridge, UKn) antibody.

ChIP-seq

Data deposition

☒ Confirm that both raw and final processed data have been deposited in a public database such as GEO.

☒ Confirm that you have deposited or provided access to graph files (e.g. BED files) for the called peaks.

Data access links<br>*May remain private before publication.*	PRJNA520738
Files in database submission	Sample_Com_7A_input<br>Sample_7A_IP<br>Sample_B_IP
Genome browser session<br>(e.g. UCSC)	https://fungi.ensembl.org/index.html

Methodology

Replicates	Two replicates.
Sequencing depth	Sample_Com_7A_input:16023405 Raw reads; single end; length : 50.88;Uniquely Mapping Ratio: 89.24%;

Sequencing depth	Sample_7A_IP: 32687065 Raw reads; single end; length: Uniquely Mapping Ratio:50.67;43.96<br>Sample_B_IP: 52144968 Raw reads; single end;length: Uniquely Mapping Ratio:50.95;42.88
Antibodies	anti-GFP ab290 (Abcam, Cambridge, UK) antibody;
Peak calling parameters	not applicable
Data quality	Sample_7A_IP: 64 peaks above 5 fold enrichment<br>Sample_B_IP: 24 peaks above 5 fold enrichment
Software	We used MACS softwere Peak Calling (http://liulab.dfci.harvard.edu/MACS/), IGV to visualize peak (http://software.broadinstitute.org/software/igv/download) and MEME to predict motif (http://meme-suite.org/).
